# Adeno-associated virus mediated artificial circular RNA for triggering cancer immunotherapy to treat prostate cancer

**DOI:** 10.3389/fonc.2025.1443571

**Published:** 2025-01-31

**Authors:** Chujin Ye, Zhiye Liu, Qifan Xie, Yanlin Tang, Jiayi Zeng, Ziwei Feng, Jiumin Liu, Haibiao Xie

**Affiliations:** ^1^ Department of Urology, Guangdong Provincial People’s Hospital (Guangdong Academy of Medical Sciences), Southern Medical University, Guangzhou, China; ^2^ The Second School of Clinical Medicine, Southern Medical University, Guangzhou, China

**Keywords:** prostate cancer, miRNA, circRNA, cancer immunotherapy, synthetic biology

## Abstract

**Introduction:**

Specifically regulating endogenous molecules is a potential molecular therapeutic strategy. Naturally occurring circular RNAs (circRNAs) are structurally stable and have been proved to serve as highly efficient miR-sponges and protein-sponges in cancer cells.

**Methods:**

We chemically synthesized circRNA (ScircRNA) *in vitro* to achieve therapeutic dysfunction by targeting specific miRNAs. RNase R and fetal bovine serum were used to evaluate the stability of ScircRNAs. In prostate cancer cell lines, the competitive inhibition of the ScircRNA on miR-375 and miR-21 activity was evaluated using luciferase report gene, cell proliferation, and apoptosis assays.

**Results:**

We found that ScircRNAs were more resistant to nuclease digestion and more effective inhibiting target miRNAs than linear RNA sponges. The ScircRNAs inhibited malignant phenotype of prostate cancer by specifically inhibiting the activity of miR-21 and miR-375. In addition, we used the ScircRNA inhibiting CDK5 expression to trigger T-cell mediated cancer immunotherapy for treating prostate cancer *in vivo*.

**Discussion:**

The ScircRNAs possessed the advantages of stable structure and simple construction, and can specifically inhibit the function of target miRNAs, which has a potential therapeutic application prospect in prostate cancer.

## Introduction

Prostate cancer (PCa) is one of the most common cancer in the world, as well as the most common tumor of the reproductive system, which is one of the most important causes of death in the world (1). Due to the molecular heterogeneity of PCa, the identification of stage-specific molecular markers is a reasonable approach that has the potential to accelerate the diagnosis, prognosis, and personalized treatment of PCa. In addition to proteins and messenger RNAs (mRNAs), microRNAs (miRNAs) are increasingly recognized for their role in regulating tumor progression.

MicroRNAs (miRNAs) are small non-coding RNAs that act through base pairing between the seed region (2-8 nucleotides) and the 3 ends of the mRNA target, which mediate the post-transcriptional silence of 30% of protein-coding genes. Abnormal miRNA transcription leads to disorder of gene expression in cells, which is an important mechanism for the formation of prostate cancer (2). Before undergoing radical prostatectomy (RP), miR-375 is also related to tumor stage and Gleason score of patients, a potential circulating marker that may be used for early detection of PCa. Recently, miR-375 purified from plasma exosomes was confirmed to be one of the biomarkers for evaluating the prognosis of castration resistant prostate cancer (CRPC) (3). In addition, miR-21 has been confirmed to be overexpressed in recurrent PCa (4). As a miRNA regulated by androgen receptor, miR-21 promotes the growth of hormone-dependent and hormone-independent prostate cancer through the p57kip2 and PTEN signaling pathways (5). In conclusion, beyond to proteins and messenger RNA (mRNAs), microRNAs (miRNAs) are increasingly recognized as biomarkers for PCa.

The circRNA belongs to a new class of non-coding RNA, which exists in a more stable circular structure rather than a typical linear structure (6). Circular RNA is processed by a special splicing mechanism in the cell. Natural circRNAs are generated by intracellular back splicing, in which the splicing donor site of the downstream exon is fused with the splicing receptor site of the upstream exon (7, 8). Because the structure of circular RNA is a closed circulation, there is no polarity at both ends of the RNA molecule, and there is no polyadenylation tail. Circular RNA is more stable than linear RNA and is not easily degraded by nucleases (9). The naturally occurring endogenous circRNA includes specific and conserved binding sites of miRNA, so that the entire circRNA acts as an effective miR “sponge” and resists miRNAs (10, 11). Recent researches of circRNA have provided new research directions for miRNAs regulation. Therefore, it is very meaningful to construct specific circular RNAs to regulate specific RNAs. In addition, in certain conditions, circRNA can be translated into peptides or proteins with essential biological functions (12). Some circRNAs had been reported to interact with important protein signaling molecules to regulate cell behaviors (13). In summary, the study of circular RNA revealed the important functions of circRNA in the physiological and pathological processes of cells, and provided a new direction for future biomedical research and therapy. The qualities of artificial synthetic circRNAs are more stable and superior, which have huge development potential, but they have been rarely explored so far.

In our study, the abnormal expression level of miR-375 and miR-21 were identified in PCa cancer cells, and ScircRNAs were used as miRNA molecular inhibitors to inhibit the growth of PCa cells. The functional unit of ScircRNA was composed of the binding sites of miR-375 and miR-21, which was designed by the sequence of miR-375 and miR-21. This ScircRNA we constructed was proved to be more resistant to nuclease degradation and more effective than the linear counterpart RNA. Next, we evaluated the competitive inhibition of miR-375 and miR-21 activities by ScircRNA and its inhibition of cell proliferation in PCa cells. We concluded that the ScircRNA was sturdy constructed, convenient, and effective genetic device to achieve miRNA-inhibition *in vitro* and has great potential for further application in therapy with PCa.

## Results

### The abnormal expression of miR-375 and miR-21 in prostate cancer cells

**Figure 1:**
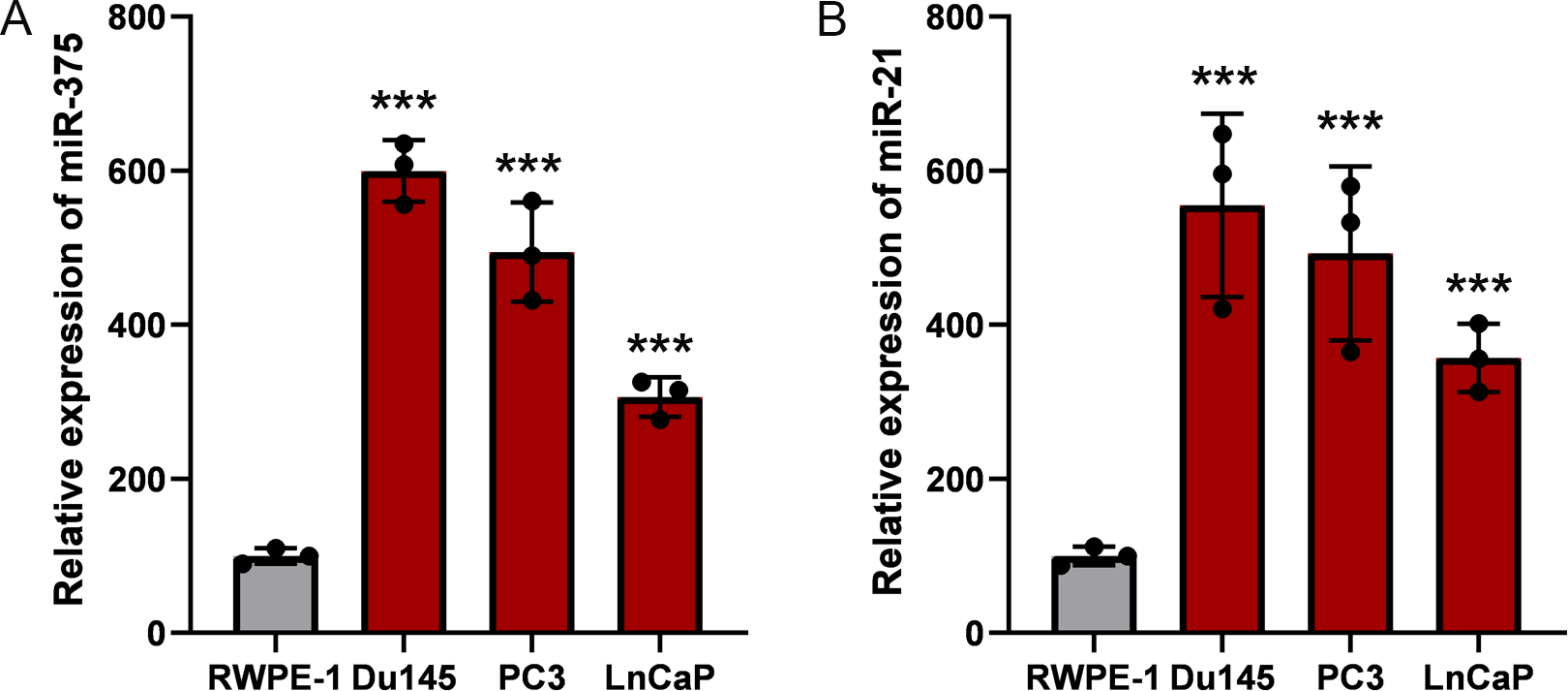
Relative expression of miR-375 and miR-21 in prostate cancer cells. The qRT-PCR assay was used to determine the expression profiles of miR-375 and miR-21 in prostate cancer cells. **(A)** The expression levels of miR-375 in RWPE-1, Du145, PC3 and LnCaP cells. **(B)** The expression levels of miR-21 in RWPE-1, Du145, PC3 and LnCaP cells. Data were exhibited as means ± s.d. from three times experiments. (***<0.001).

To investigate the expression profile of miR-375 and miR-21, the qRT-PCR assay were performed to identify the expression level of prostate cancer cells (LNCAP, Du145, PC3 and C4-2) compared with the normal prostate epithelial cell (RWPE-1). We found that miR-375 showed the highest expression level in Du145 cell, followed by LNCaP and PC3 cells, and miR-375 showed the relatively lower expression level in C4-2 cells (**Figure 1A**). The expression of miR-21 was the highest in PC3 cells, the lowest in C4-2, and the expression levels of Du145 and LNCaP cells were between the former two (**Figure 1B**). Due to both miR-375 and miR-21 were relatively over-expression in Du145 and PC3 cells, we chose these two types of prostate cancer cells for the following studies.

### The design and construction of synthetic circular RNA

**Figure 2:**
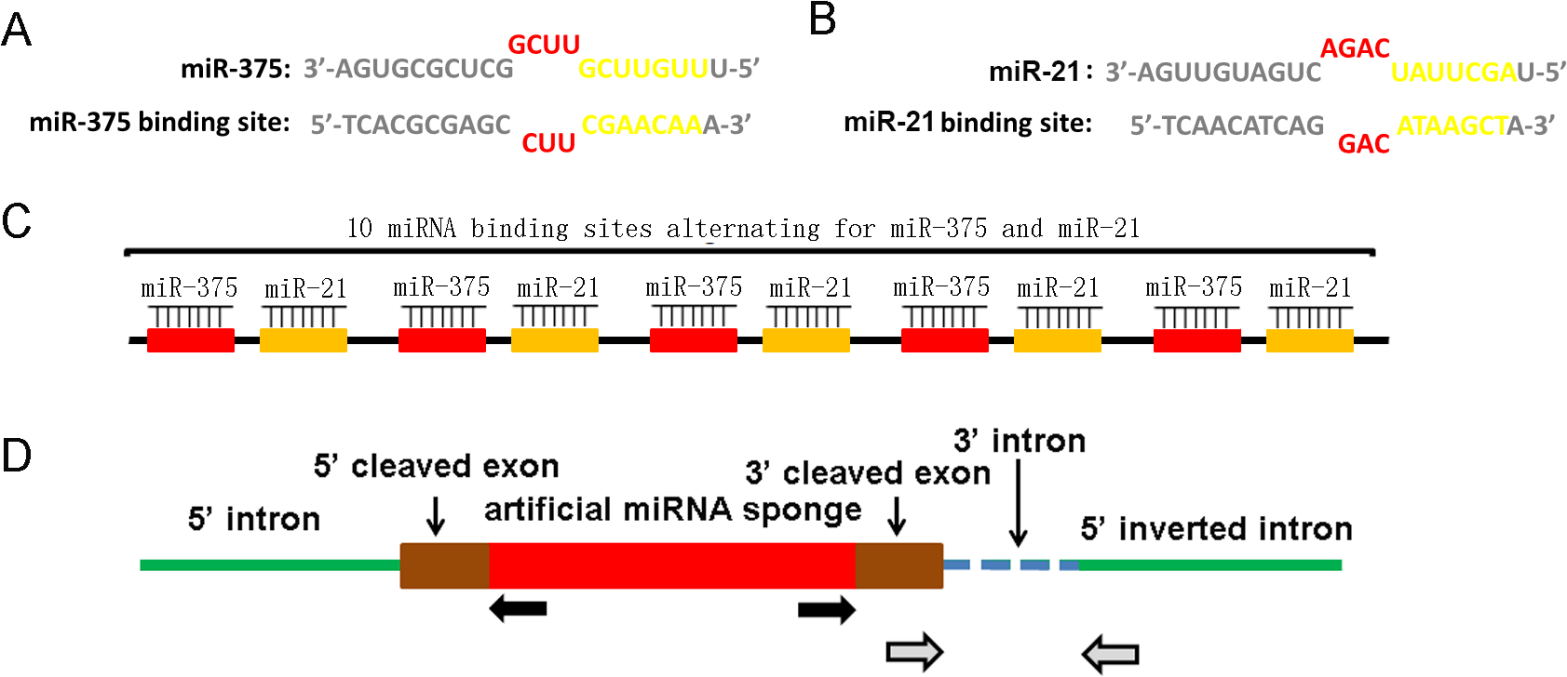
Design and construction of ScircRNA. **(A)** The miRNA binding sites of miR-375 and miR-21 were taken the imperfect complementary design rather than a perfectly complementary design. One single base was deleted at the 9-12 nt position of the miRNA sequence together with three base mismatches resulted in a bulge of miRNAs binding sequences. The seed areas were highlighted in blue. The sequence and binding site of miR-375 were shown in the left, while those of miR-21 were exhibited in the right. **(B)** The schematic of the basic structure of ScircRNA. Each ScircRNA contained 10 miRNA binding sites, and each individual binding site was separated by 6 nt spacers. **(C)** The expression pattern of ScircRNA was shown in the schematic diagram. The gray arrow showed the location of RNA aggregation sites, while the black arrow showed the location of circrRNAs specific differentiation sites.

ScircRNAs we designed inhibit the abnormally over-expression of miRNAs in prostate cancer cells by specific sponge of intracellular miRNAs. Structurally, the functional regions of ScircRNAs are mainly composed of the specific binding sites of miR-375 and miR-21. The binding site we designed did not perfectly match the target miRNA sequence, while deleted one base from the 9th to the 12th base and introduced three mismatches. This design would produce bulged binding sites in the corresponding region, which is more conducive to the binding of target miRNA to the binding site (14, 15). The binding sites of miR-375 and miR-21 were engineered as illustrated in **Figure 2A** and **Figure 2B**. One miR-375 binding site and one miR-21 binding site constituted a unit of ScircRNA, while there are 5 units forming one ScircRNA. These designs are based on previous optimization studies on linear miRNA sponge designs.

The *in vitro* cyclic strategy for ScircRNAs is to synthesize linear RNAs using enzyme ligations, which contain five repeating units protrusion binding sites (15) (**Figure 2C**). The accuracy of the ScircRNA construction was confirmed by Sanger sequencing. To cyclization the linear sequence of ScircRNA, the inverted complementary introns were added to both sides of the sequence, which would cause the ends of the sequence to automatically join into a loop (**Figure 2D**).

### Synthetic circRNAs are more stable than synthetic linear counterpart RNAs

**Figure 3:**
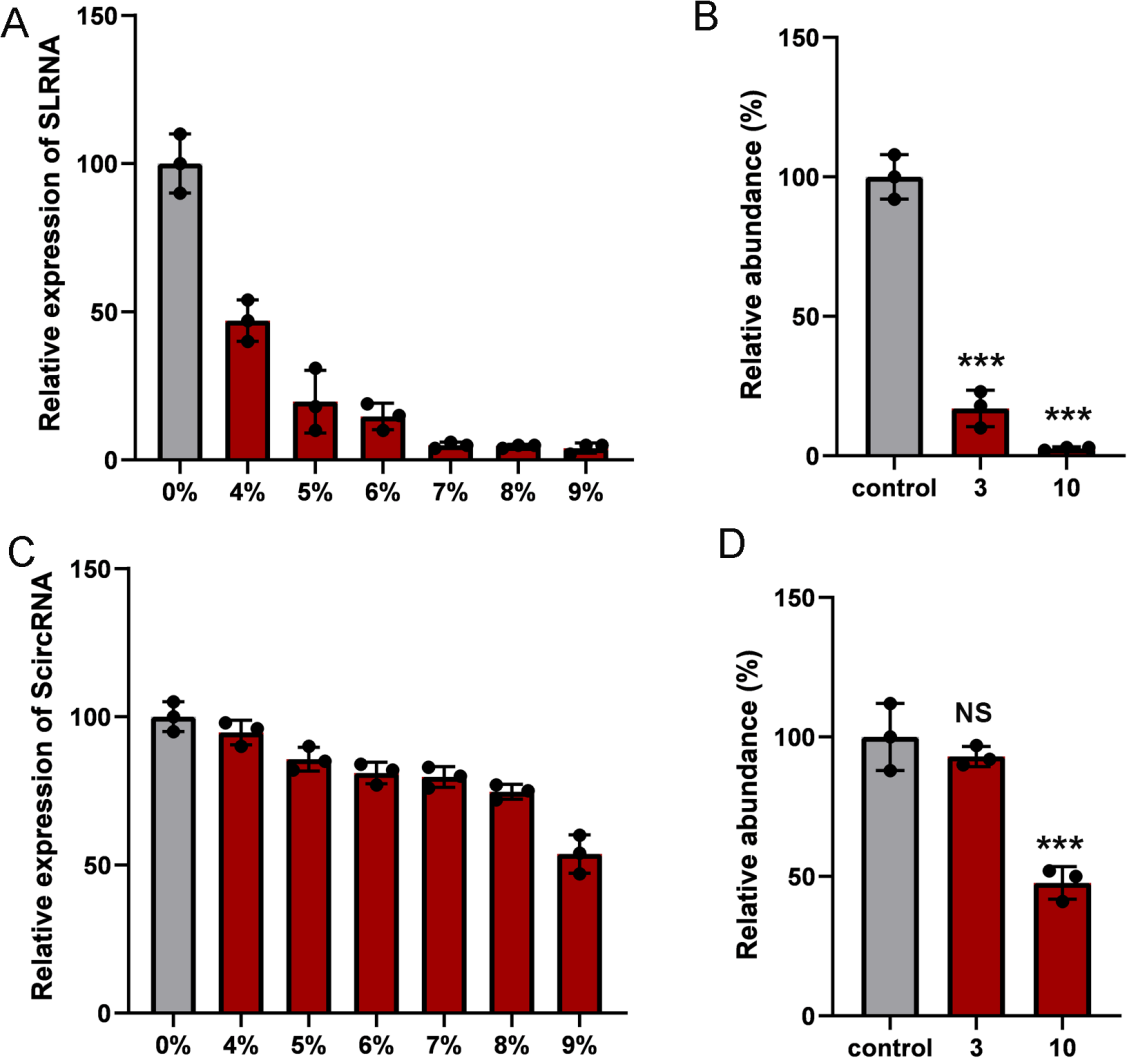
The stability of ScircRNAs. Nuclease digestion was used to determine the resistance of ScircRNAs. **(A)** The relative expressions of SLRNA were determined by increasing concentrations of fetal bovine serum at 37°C for 30 min. **(B)** The resistances of SLRNA were measured in 0 ug, 1ug and 3ug of RNase R. **(C)** The relative expressions of ScircRNA were determined by increasing concentrations of fetal bovine serum at 37°C for 30 min. **(D)** The resistances of ScircRNA were measured in 0 ug, 1ug and 3ug of RNase R. Data were exhibited as means ± s.d. from three times experiments. (NS, not significant; ***<0.001).

We evaluate the stability of RNA using fetal bovine serum (FBS) and the specific exonuclease, RNase R. Theoretically, the circular structure would protect the ScircRNAs themselves from degradation. Stability of ScircRNAs was determined compared with their linear counterpart RNA (SLRNA), and first using FBS-mediated degradation. We set different concentrations of FBS (from 4% to 9%), and incubate ScircRNA and SLRNA in FBS for 30 minutes at 37°C. The qRT-PCR assay was used to determine the degradation levels of ScircRNA and SLRNA, respectively. SLRNA showed 82% degradation (**Figure 3A**), while ScircRNA only showed 5% degradation (**Figure 3C**). In fact, when the concentration of FBS is above 9%, ScircRNA showed more obvious degradation. In addition, we used specific exonuclease RNase R to compare the stability of circular and linear RNAs. The results showed that the SLRNA was 92% degraded in 3 U/mg of RNase R (**Figure 3B**), while the ScircRNA was only 10% degraded (**Figure 3D**).

### Synthetic circRNAs specifically inhibiting the expression of miR-375 and miR-21

**Figure 4:**
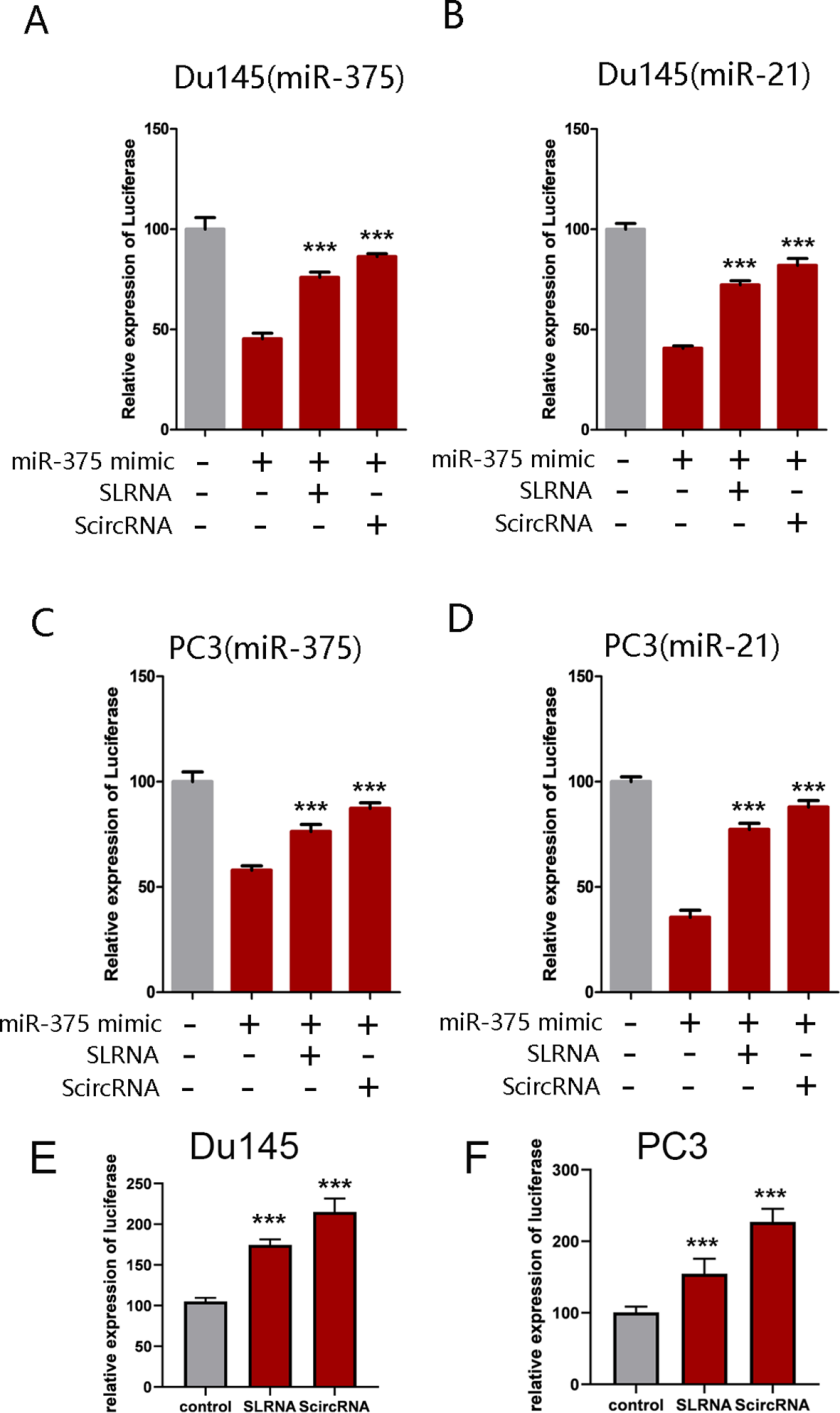
Effective of ScircRNA serving as a miRNA sponge. Dual-luciferase reporter assay was performed to determine the effective of ScircRNA acting as a miRNA sponge compared with SLRNA. **(A)** The effective of ScircRNA inhibiting miR-375 in Du145 cells. **(B)** The effective of ScircRNA inhibiting miR-21 in Du145 cells. **(C)** The effective of ScircRNA inhibiting miR-375 in PC3 cells. **(D)** The effective of ScircRNA inhibiting miR-21 in PC3 cells. **(E)** The effective of ScircRNA inhibiting endogenous miR-375 and miR-21 in Du145 cells compared to the SLRNAs. **(F)** The effective of ScircRNA inhibiting endogenous miR-375 and miR-21 in PC3 cells compared to the SLRNAs. Data were exhibited as means ± s.d. from three times experiments. (*<0.05, **<0.01, ***<0.001). Data were exhibited as means ± s.d. from three times experiments. (***<0.001).

The binding sites of miR-375 and miR-21 were inserted into the 3’ untranslated region of the renilla luciferase to construct the miR-375 and miR-21 dual-luciferase reporter system, respectively. As expected, co-transfection of the luciferase reporter systems with miR-375 and miR-21 mimics into Du145 and PC3 cells can significantly reduce luciferase activity. Upon introduction of ScircRNAs or SLRNAs, luciferase activity was significantly rescued, in which the binding sites of miR-375 and miR-21 were scrambled (**Figure 4**). In addition, we found that group ScircRNA showed more obvious reversal effect of luciferase expression than group SLRNA, indicating that the ScircRNA with more stable structure exhibited better binding efficiency to target miRNA. Next, we identified the inhibition of ScircRNA on miR-375 and miR-21 in primary prostate cancer cells. Prostate cancer cells Du145 and PC3 were selected to test the inhibitory effect of ScircRNA. As expected, ScircRNA also promoted the expression of luciferase in prostate cancer cells, which was achieved by disrupting the binding of the endogenous miR-375 and miR-21 to the binding sites of luciferase reporter systems. The results also showed that ScircRNA had a better inhibitory effect of miR-375 and miR-21 than SLRNA in prostate cancer cells.

### Synthetic circRNAs inhibiting malignant phenotypes of prostate cancer cells effectively

**Figure 5:**
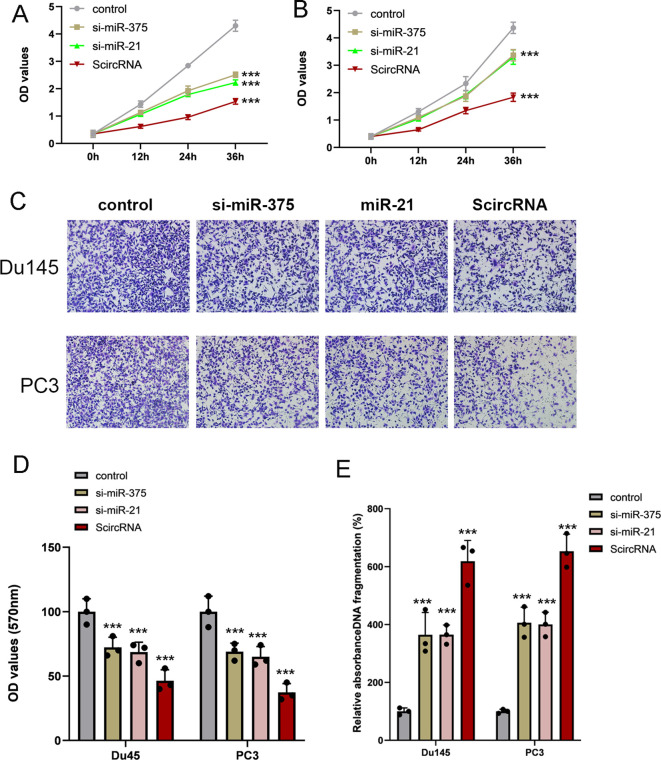
Inhibition of malignant phenotype by ScircRNAs in prostate cancer. **(A)** Compared with siRNAs of miR-375 and miR-21, ScircRNA shown the stronger inhibition of cell proliferation in Du145 cells. **(B)** Compared with siRNAs of miR-375 and miR-21, ScircRNA shown the stronger inhibition of cell proliferation in PC3 cells. **(C)** Representative transwell images of each groups. Scale bar 100μm **(D)** All the migration data were shown as means ± s.d. from triplicate experiments. **(E)** Cell death ELISA was performed to determine the cell apoptosis in prostate cancer. ScircRNA showed significant inhibition of cell invasion in Du145 cells and PC3 cells. Data were exhibited as means ± s.d. from three times experiments. (NS, not significant; ***<0.001).

As oncogenic miRNAs, miR-375 and miR-21 are highly expressed in prostate cell lines and affect the malignant progression of tumors. Therefore, we determined the effect of ScircRNA on suppressing malignant phenotype of PCa. To identify that ScircRNAs would inhibit PCa in all stages, PC3 cells and Du145 cells were selected in our study. The CCK-8 assay was performed to determine cell proliferation suppressed by ScircRNAs, and nonfunctional artificial circRNAs were designed to serve as control groups. As shown in the **Figures 5A, B**, ScirRNA significantly inhibited cell proliferation of PCa cells. In addition, we further determined the effects of ScircRNAs on the cell invasion and cell apoptosis of PCa using the transwell assay and ELISA assay, respectively. We found that ScircRNAs could significantly inhibit cell invasion of PCa (**Figures 5C, D**), as well as the malignant phenotype of apoptosis (**Figure 5E**).We concluded that ScircRNAs exerted their inhibition function in PCa cells. In addition, we found that compared with linear RNA sponge for miR-375 (si-miR-375) or miR-21 (si-miR-21), ScircRNA showed the stronger inhibitory effect on malignant phenotypes of prostate cancer.

### Potential to inhibit malignant phenotype of prostate cancer using AAV mediated ScircRNA

**Figure 6:**
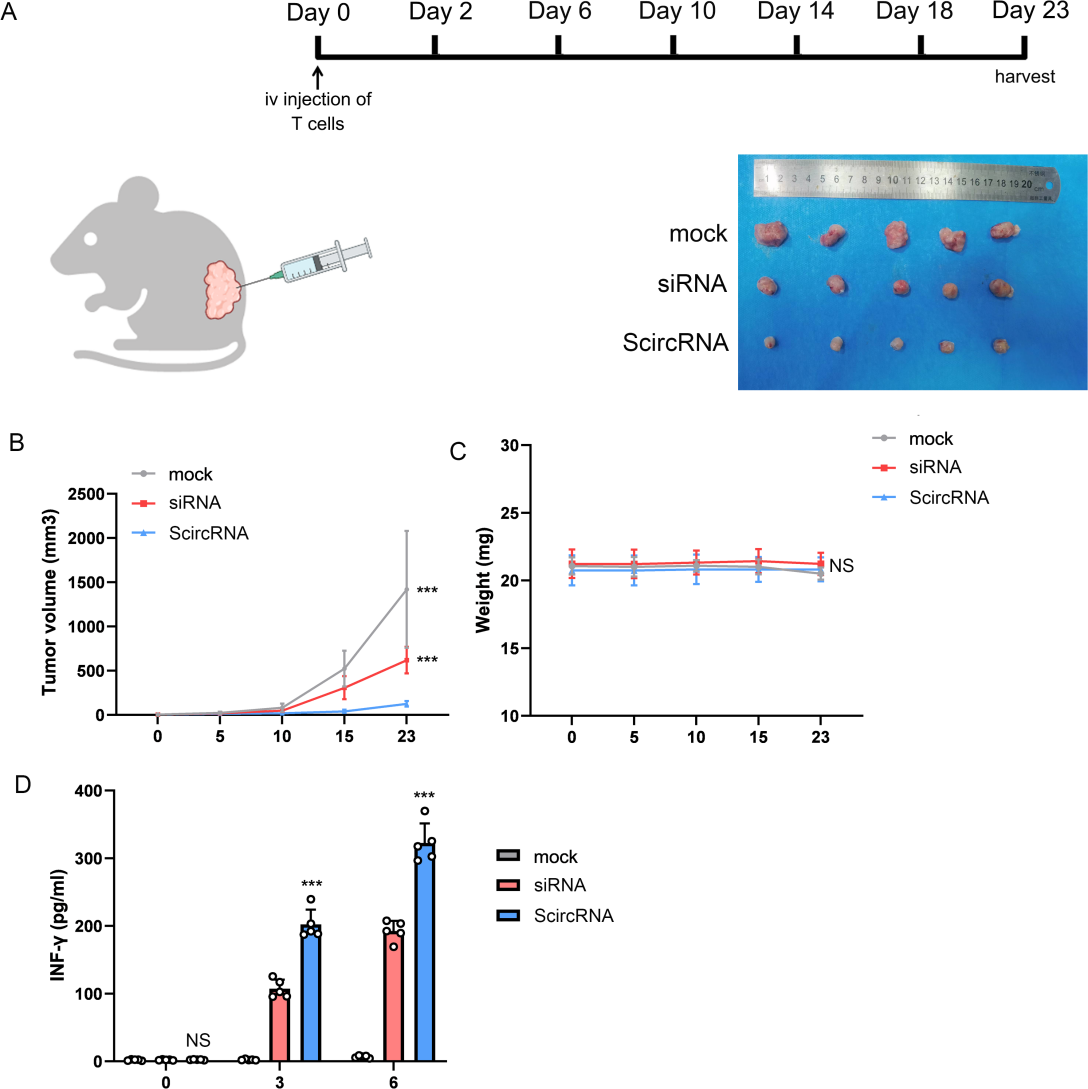
The effect of ScircRNA mediated cancer immunotherapy killing cancer cells *in vivo*. **(A)** The mouse immunotherapy regimen mediated by AAV suspension loaded with gene circuit. **(B)** The tumor volume change of subcutaneous transplanted tumor in each group. **(C)** The weight change of each group. **(D)** The changes of INF-γ in blood of mice in each group. (NS, not significant; ***<0.001).

To investigate the *in vivo* therapeutic effect of the ScircRNA system, we implemented a human xenograft tumor mouse model to explore the role of ScircRNA in T-cell-mediated immunotherapy for specific anti-tumor efficacy. Here, we designed the ScircRNA to express the specific siRNA for inhibiting the Cyclin-dependent kinase 5 (CDK5) expression, which would inhibited the expression of interference on regulatory factor 2 to down-regulate the expression level of PD-L1. We activated tumor immunity in this way to treat prostate cancer. The control tumor xenograft and target tumor xenograft were prepared by subcutaneously implanting into the NSG mice (**Figure 6A**). The human primary T cells were infused intravenously into the tumor-bearing NSG mice after forming tumors for 3 days. Next, different AAV suspensions were intratumorally injected at day 0 and the tumor volumes were harvested in Day 23 (**Figure 6A**). Tumor volume growth was significantly inhibited in the infusion treated mice compared with the control group that received T cells alone (**Figure 6B**). We also found that the mice treated with the AAV suspension included antibody system and T cells shown the best inhibitory effect and the T cells activation level (**Figure 6D**).

## Discussion

Prostate cancer (PCa) is among the most common forms of cancer affecting males throughout the world. Human PCa development occurs through a multi-stage process that culminates in the metastasis of these tumor cells to distant sites including the liver, lungs, and bone. Initially, prostate tumors grow in an androgen-dependent manner, although they ultimately enter into an androgen-independent growth stage through mechanisms that are incompletely understood, thus giving rise to castration-resistant prostate cancer (CRPC), which renders patients insensitive to hormone therapy and is prone to readily undergoing distant metastasis (16). Treatments developed for PCa include combinations of surgery, chemotherapy, and immunotherapy, with each exhibiting specific advantages and disadvantages (17). Therefore, to treat PCa, especially CRPC, it is urgent to explore some more effective manners.

The miRNAs are important regulators that regulate gene expression at the level of gene transcription. They base pair the binding sequence of the target mRNA, which will cleave the target mRNAs to inhibit translation and thereby inhibit the expression of the target genes (18). There was growing number of strategies being developed to implement the loss of miRNA functionality. The RNA molecular sponges containing binding sites which are complementary to miRNAs of interest are designed to regulate cell phenotype (14). For example, the artificial synthetic long non-coding RNA which serviced as the miRNA sponge was designed to inhibit miR-183, miR-182, miR96 and miR-17-5p to inhibit bladder cancer (19). There was no doubt that the miRNA molecular sponges have huge development potential in the direction of tumor treatment. However, the design of traditional molecular sponge of miRNA is based on linear structure, and there are unavoidable shortcomings such as structural instability. The structural instability of linear miRNA molecular sponges affects their inhibitory effect on miRNA, which also limits the further use of linear molecular sponges.

The naturally occurring circular RNAs have the two characteristics of exonuclease resistance and conserved miRNA binding sites, which have caused them to receive extensive attention (20). The emerging evidences showed that the naturally occurring circRNAs were effective miRNA sponges and played important regulatory roles in the pathogenesis of cancer. For example, endogenous cicRNS-7 contains multiple conserved miR-7 binding sites, which makes the entire circRNA-7 function as a natural sponge of miR-7. The overexpressed siRS-7 activated the downstream oncogenes EGFR and RAF1 of miR-7 by inhibiting miR-7, which made colorectal cancer more aggressive (21).

The synthesis of circRNA was constructed by *in vitro* synthesis, which combined the binding sites of miRNAs through enzyme ligation (15). As shown in our results, compared with the linear RNA molecular sponge, the circRNAs we constructed due to their circular structure, which made the entire RNA molecular structures have no free 5’ and 3’ ends, was more resistant to nuclease digestion. In addition, we found that compared with linear miRNA sponge, circRNA showed a better inhibitory ability of miRNAs. This may be because circRNA is more stable than linear RNA in the intracellular environment and exists for a longer time. We combined the binding sites of different miRNAs (miR-375 and miR-21) into one functional units of circRNA which would achieve the co-inhibition of different miRNAs to enhance the inhibitory effect on prostate cancer cells. Notably, previous studies had reported that CDK5 negatively regulated the expression level of PD-L1 protein (22). In other word, inhibition of CDK5 can significantly up-regulate the expression level of PD-L1 in tumors and improve the efficacy of T-cell-mediated tumor immunotherapy. Targeting miR-21 had been reported to regulate CDK5 expression in head and neck squamous cell carcinoma (23). In addition, several papers had demonstrated the relationship between the high expression of miR-375 and PD-L1, and clarified that miR-375 is a potential target for tumor immunotherapy (24–26). Therefore, inhibition of miR-375 and miR-21 may be a novel immunotherapy strategy. The inhibition of these two miRNAs by ScircRNA described in this paper to enhance T-cell-mediated tumor immunity may have some reference value for the development of immunotherapy for prostate cancer.

The methods of treating cancer based on miRNA inhibition should be to show high miRNA inhibitory activity under the premise of low dose, and to exist as stably as possible. Circular RNA possesses the important property of resisting nuclease degradation without chemical modification. Our experimental results also showed that circRNA have the potential to prolong miRNA inhibition time, which also indicates that circRNAs require only a minimum dose or longer dosing interval. Therefore, compared with the linear miRNA molecular sponges, circRNAs have more potential for development in the cancer treatment by regulating miRNAs.

## Methods

### Cell lines and cell culture

Human prostate cancer cell lines LNCAP, DU145 and PC3 were purchased from the Culture bank of the United States (Manassas, VA, USA). All cell lines were cultured in DMEM (GIBCO, Grand Island, NY, USA) supplemented with 1% penicillin-streptomycin (GIBCO) and 10% fetal bovine serum (GIBCO). The petri dishes were cultured in a humidified atmosphere of 37°C and 5% CO2.

### RNA extraction and quantitative real-time PCR

TRIzol reagents (Invitrogen, Carlsbad, CA, USA) were used to extract the total RNA in our work. The cDNA required for the experiment was obtained by a reverse transcription experiment, which was performed by the Prime-Script RT reagent kit with gDNA Eraser (TaKaRa, Japan). Real-time quantitative PCR was performed using SYBR Premix Ex Taq II (TaKaRa, Japan) and 7500 Fluorescence Quantitative PCR system (Applied Biosystems Life Technologies, USA). The data results were normalized to β-actin or U6 small nuclear RNA.

### Circular sponge design

Each mir-375 binding site we designed did not completely complement the Mir-375 seed region, but instead deleted one nucleotide, which resulted in a bulge (mismatch) at from the ninth to the twelfth base of Mir-375 (14). The sequence of miR-375 binding site was 5’-TCACGCGAGCCUUCGAACAAA-3’, while the binding site sequence of miR-21 was 5’-TCAACATCAGGACATAAGCTA-3’. Four blank bases (CCAA) were inserted between each individual binding site, which also called spacer region.

The RNA sponge composed of scrambled binding site sequences was used as a control group in our work. The miR-375 scrambled binding site sequence was 5’-TGGCTACCTAATCGCAATGCC-3’, while the sequence of miR-21 scrambled binding site was 5’-ACTTAGTGTCTATGCGTATGT-3’.

### RNase R digestion

Ribonuclease is used to evaluate the circularity of synthetic circular RNA. We add scRNA and LRNA with or without RNase R (Epicenter Biotechnologies) and incubate at 37°C for 10 minutes. Then the qRT-PCR assay was used to evaluate the stability of the synthesized RNA molecules.

### Luciferase reporter assay

The binding sites of miR-21 and miR-375 were inserted into the pmirGLO vector (Promega, Madison, WI, USA) using PmeI and XbaI restriction enzymes (New England Biolabs) to construct the miR-21 and miR-375 luciferase reporter vectors which containing miR-21 and miR-375 binding sites respectively. 24 h after transfection with the corresponding vector, the luciferase activity of the cells was analyzed using the dual luciferase assay kit (Promega) and VICTOR2 fluorometric assay (Perkin Elmer, Waltham, MA, USA). Firefly luciferase activity is normalized to renal cell luciferase activity. All the assays were repeated at least three times.

### CCK-8 assay

The cell proliferation of PCa cells was determined using CCK-8 assay. The specific experimental operations refer to previous studies (27). The absorbance of each well was determined by a microplate reader (Bio–Rad, USA) at 0, 12, 24, 36, and 48 h.

### Transwell assay

The transwell assay was used to determine the level of cell invasion. 5 × 10^4^ cells were inoculated in serum-free medium in the upper chamber and medium containing 10% FBS in the lower chamber. After 24 h incubation, the cells remaining in the upper chamber were cleared, and the cells migrating to the bottom were fixed with 4% paraformaldehyde and imaged.

### ELISA assay

Cell apoptosis was determined by quantification of histone complex DNA fragments in cytoplasm by cell death assay ELISA (Roche Applied Science). The Microplate reader (Bio-RAD) was used to determine the absorbance of DNA fragments at 405 nm wavelengths. The experiment was repeated independently for 3 times for each sample.

### AAV packaging, purification and virus titer detection

HEK-293T cells were transfected with pAAV packing plasmid, pHelper plasmid and pAAV plasmid using Lipofectamine 3000. The culture supernatant was collected 48 hours after transfection and concentrated as a viral reservoir for subsequent AAV infection experiments. AAV titers were calculated using qPCR method of 2× EvaGreen Master Mix (Syngentech).

### Tumor xenotransplantation

All mice were housed in standard laboratory conditions. BALB/c nude mice were randomly divided into experimental group and control group (5 mice in each group). PCa cells (10^8^) were injected subcutaneously into the back of BALB/c-Nude mice. AAV was then injected. The calculation formula for tumor volume is V = L×W^2^/2, where L is the length of the tumor and W is the width of the tumor. At the end of the experiment, the mice were harvested and their tumors removed.

### Statistical analyses

All the experimental data were analyzed using GraphPad Prism and SPSS 19.0 (IBM, SPSS, Chicago, IL, USA). The student’ *t-test* or *ANOVA* were used for inter-group differences. A P value < 0.05 was considered statistically significant.

## Data Availability

The datasets presented in this study can be found in online repositories. The names of the repository/repositories and accession number(s) can be found in the article/supplementary material.
